# Tracking the intrusion of unwanted memories into awareness with event-related potentials

**DOI:** 10.1016/j.neuropsychologia.2016.07.008

**Published:** 2016-08

**Authors:** Robin Hellerstedt, Mikael Johansson, Michael C. Anderson

**Affiliations:** a Department of Psychology, Lund University, Paradisgatan 5 P, 223 50 Lund, Sweden; b MRC Cognition and Brain Sciences Unit, University of Cambridge, 15 Chaucer Road, Cambridge CB2 7EF, UK

**Keywords:** Intrusive memory, Involuntary retrieval, ERP, Think/No-Think, Episodic memory, Forgetting

## Abstract

Involuntary retrieval of unwanted memories is a common symptom in several clinical disorders, including post-traumatic stress disorder. With an aim to track the temporal dynamics of such memory intrusions, we recorded electrophysiological measures of brain activity while participants engaged in a Think/No-Think task. We presented the left hand word (the cue) of previously encoded word pairs in green or red font. We asked participants to think of the associated right hand word (the associate) when the cue appeared in green (Think condition) and to avoid thinking of the associate when the cue appeared in red (No-Think condition). To isolate cases when participants experienced an intrusive memory, at the end of each trial, participants judged whether the response had come to mind; we classified memories that came to mind during No-Think trials, despite efforts to stop retrieval, as intrusions. In an event-related potential (ERP) analysis, we observed a negative going slow wave (NSW) effect that indexed the duration of a trace in mnemonic awareness; whereas voluntary retrieval and maintenance of the associate was related to a sustained NSW that lasted throughout the 3-s recording epoch, memory intrusions generated short-lived NSWs that were rapidly truncated. Based on these findings, we hypothesize that the *intrusion-NSW* reflects the associate briefly penetrating working memory. More broadly, these findings exploit the high temporal resolution of ERPs to track the online dynamics of memory intrusions.

## Highlights

• We tracked the intrusion of unwanted memories into awareness with ERPs.
• Memory intrusions were related to an FN400, a NSW and a reduced LPP effect.
• The NSW effect tracked people’s awareness of an intrusive memory.

## 1 Introduction

There are life experiences that we would rather forget. Often, these episodes are particularly well encoded in long-term memory, and when confronted with a reminder of the unwanted memory we may retrieve it involuntarily. Such memory intrusions are common in the general population (e.g. Rachman and de Silva, 1978) and are also a key symptom of posttraumatic stress disorder (American Psychiatric Association, 2013). Memory intrusions have been related to several other psychiatric disorders including depression (e.g. Brewin et al., 2010, Reynolds and Brewin, 1999) and social phobia (e.g. Hackmann et al., 2000). The present study leveraged high temporal-resolution electroencephalography (EEG) to track the dynamics of memory intrusions.

Neuroimaging studies of memory retrieval have thus far mainly focused on voluntary retrieval. The little research that has examined involuntary retrieval has concentrated on incidental retrieval rather than on memory intrusions. Incidental retrieval refers to cases in which memories are accessed unintentionally. Incidental retrievals indicate that the retrieval process does not require explicit intention to be engaged, but they do not clearly establish whether retrieval is truly involuntary. In the present study, we use the term *memory intrusion* to refer to retrievals that are not merely unintentional, but that are *counter-intentional*. That is, intrusions are memories that are retrieved, despite efforts to prevent retrieval from occurring, providing a clear operational definition of involuntary access.

Memory intrusions can be studied experimentally with the Think/No-Think paradigm (Anderson and Green, 2001; Anderson & Huddleston, 2012; Anderson and Levy, 2009, Anderson et al., 2004). In this paradigm, participants learn word pairs before engaging in a Think/No-Think task. In the Think/No-Think task, the left hand word (the cue) typically appears in green or red font colour. The participants are instructed to think of the associated right hand word (the associate) when a cue word is shown in green (Think condition) and to avoid thinking of the associate when the cue is presented in red (No-Think condition). A surprise cued recall test is given in the final part of the paradigm. The typical finding in this test is that repeated retrieval suppression attempts cause forgetting in the No-Think condition compared with word pairs that were not included in the Think/No-think task (behavioural baseline condition); i.e. intentional forgetting (for a review see Anderson and Huddleston, 2012). This finding is referred to as *suppression-induced forgetting* (Anderson and Hanslmayr, 2014).

We modified the Think/No-Think paradigm in two ways to investigate the event-related potential (ERP) correlates of memory intrusions. First, immediately following each Think or No-Think trial, we asked participants to report whether the associate had entered awareness at all (cf. Benoit et al., 2014, Levy and Anderson, 2012). We used this introspective report to divide the No-Think condition into memory intrusion and successfully avoided retrieval trials in our ERP analysis. Second, we added a perceptual baseline condition for use in the ERP analyses. This condition consisted of trials presenting single words that had been presented among the word pairs in the learning phase of the experiment. These single words appeared in yellow font during the Think/No-Think phase and participants simply read these words and kept them in mind until the end of the trial. The intention of this baseline is to provide a way of measuring ERPs generated by simply viewing an episodically familiar word that does not have an associated response stored in memory. A recent EEG study introduced a similar baseline, although with a different data analysis approach (Depue et al., 2013).

To track the temporal dynamics of memory intrusions, we analysed the ERP data according to the following logic: First, we characterized the ERP correlates of voluntary retrievals and of memory intrusions by comparing each of these two conditions with the perceptual baseline. Next, we sought to isolate differences between voluntary retrievals and memory intrusions. Finally, we compared memory intrusions with avoided retrievals. This final comparison allowed us to match the characteristics of the No-Think task that may not be involved in our perceptual baseline, and to isolate the dynamics of intrusive memories.

Previous research has established ERP correlates of multiple cognitive processes involved in voluntary and incidental memory retrieval. Given the lack of prior knowledge regarding the ERP correlate of memory intrusions, we assumed that they would be reflected in one or more of the ERP effects related to voluntary retrieval. This assumption is partly based on studies of incidental memory retrieval that have shown that, although there are neural correlates specific for incidental retrieval, considerable overlap exists between the neural mechanisms involved in incidental and voluntary memory retrieval (Curran, 1999, Hall et al., 2014, Kompus et al., 2011, Rugg et al., 1998). Three candidate long-term memory retrieval ERP effects are described below.

The earliest ERP effect likely to be involved in memory intrusions is the FN400. This ERP effect is an attenuation of a negative slow wave which onsets approximately 300 ms after cue presentation and is maximal over mid anterior regions. The FN400 effect has been related to recognition of a probe without retrieval of contextual details from the encoding episode, i.e. familiarity (for review see Rugg and Curran, 2007), and to facilitated access to semantic memory representations that are related to the presented probe after repeated exposure, i.e. conceptual priming (for review see Paller et al., 2007). Of importance for the present cued recall study, the FN400 has been related to the reactivation of paired associates in cued recall (Hellerstedt and Johansson, 2014) and recognition tasks (Opitz and Cornell, 2006). In the present study, all cues except the ones in the perceptual baseline condition were paired with an associate in the encoding phase. Consequently, we expected both voluntary retrieval and memory intrusions to be reflected in an FN400 compared with the perceptual baseline. The FN400 has not only been related to reactivation of associated memories, but also to interference and subsequent forgetting of the reactivated memories (Hellerstedt and Johansson, 2014). Thus, in the No-Think condition we expected the FN400 to reflect the reactivation of the to-be-avoided memory, indicating the need to suppress retrieval during memory intrusion trials (i.e. activation of the associate).

Another ERP effect implicated in long-term memory retrieval is the left parietal positivity (LPP; Paller and Kutas, 1992, Smith, 1993, Wilding and Rugg, 1996). This ERP effect has been related to vivid retrieval of details from the encoding situation, i.e. recollection (Mandler, 1980) in both recognition (for review see Rugg and Curran, 2007) and cued recall (for review see Friedman and Johnson, 2000) paradigms. The LPP is a positive going modulation onsetting approximately 500 ms after the presentation of the test probe and is maximal over left posterior regions (Friedman and Johnson, 2000, Rugg and Curran, 2007). Of importance for the present study, this effect has also been shown in incidental retrieval (Curran, 1999, Kompus et al., 2011), suggesting that it is independent of retrieval intention. Interestingly, retrieval suppression attempts in the Think/No-Think paradigm have been related to a reduction of the LPP effect (Bergström and de Fockert, 2009, Bergström et al., 2009, Bergström et al., 2007, Chen et al., 2012, Depue et al., 2013, Hanslmayr et al., 2009, Mecklinger et al., 2009, Waldhauser et al., 2012). An interpretation of this finding is that recollection can be avoided via retrieval suppression. We expected that both voluntary retrieval and memory intrusions would yield an LPP effect compared with the perceptual baseline.

A third ERP effect that may be related to memory intrusions is the negative slow wave (NSW). This effect has been related to working memory maintenance (for reviews see Drew et al., 2006, Ruchkin et al., 2003). In the present study, we instructed participants to think of the retrieved associate until the end of the trial in the Think condition. Consequently, we expected a NSW late in the recording epoch (after retrieval) when contrasting voluntary retrieval and memory intrusions with the perceptual baseline. Working memory maintenance of verbal stimuli has been related to a left anterior NSW both in the visual and the auditory domain (e.g. Ruchkin et al., 2003). Moreover, the NSW has also been observed in episodic long-term memory experiments like the present study, where a retrieved memory representation needs to be held in working memory until the response is given (e.g. Khader et al., 2005, Rösler et al., 1995). Critically, because we instructed the participants to purge the associate out of awareness as soon as they noticed a memory intrusion, we expected the NSW to have a shorter duration for memory intrusions compared with voluntary retrievals.

Finally, we expected to replicate other ERP effects related to additional cognitive processes involved in the Think/No-Think task, like the P2 effect that has been suggested to reflect allocation of attention to the colour features of the cue and task selection based on this information (Bergström et al., 2007, Mecklinger et al., 2009, Waldhauser et al., 2012) and the N2 effect that has been related to cognitive control (Bergström et al., 2009, Mecklinger et al., 2009, Waldhauser et al., 2012).

## 2 Methods

### 2.1 Participants

Thirty-six right-handed, native Swedish speakers, with normal or corrected to normal vision, including normal colour vision, gave written informed consent before participating in exchange for two cinema tickets. All participants reported no history of psychiatric or neurological disorders. We excluded four participants that reported too few intrusions or avoided retrievals in the Think/No-Think phase of the experiment to enable ERP analysis. The final sample consisted of 32 participants (12 men, mean age = 25 years, range = 20–34 years). The study had been approved by the regional ethics committee at Lund University.

### 2.2 Materials

The stimuli consisted of 96 semantically unrelated Swedish word pairs. The word pairs were divided into eight lists. Two lists (24 word pairs) were assigned to each of the three conditions that were included in the Think/No-Think phase of the experiment (i.e. the Think, the No-Think and the perceptual baseline condition). One list (12 word pairs) was assigned to the behavioural baseline condition and the word-pairs in the final list served as fillers (12 word pairs). The lists were matched for word length and word frequency. The assignment of lists to conditions was counterbalanced across participants.

### 2.3 Procedure

The experiment consisted of three phases: study, Think/No-Think and test. The study phase was divided into three sub-phases (presentation phase, test-feedback phase and criterion test phase). In all phases of the experiment stimulus presentation was preceded by a fixation cross (shown for 500 ms) and a black screen (displayed for 250 ms in the study phase and 500 ms in the Think/No-Think phase and the test phase).

#### 2.3.1 Study phase

The study phase was divided into four blocks to facilitate learning of the stimulus material. In each block, 18 word pairs were presented in random order in white on black background for 3000 ms. We told a cover story to the participants to make sure that they tried to suppress retrieval later in the Think/No-Think task. We told them that they were about to participate in an attention experiment and that they were going to learn word pairs that would be used in the attention experiment later. We instructed the participants to form an association between the words so that they could recall the associate when given the cue later in the experiment. In addition to the 18 word pairs, six single words were presented on the left side of the screen. We instructed the participants to learn the single words and used them as a perceptual baseline in the ERP analysis. A feedback-cued-recall test followed when all the word pairs and single words had been presented. In this test, the participants were provided with the left hand word (the cue; displayed for 3000 ms) and were asked to recall the right hand word (the associate). The correct associate was displayed in blue font after the response for 1000 ms. We instructed the participants to read the cue word aloud when a single word appeared on the screen to indicate that they had learned that this was a single word. The feedback-cued-recall test continued until the participants had learned more than two thirds of the word pairs. When the learning criterion had been met in all four blocks of items the participants engaged in a criterion cued recall test, without feedback. We restricted the analyses to learned items, as indicated by performance in this test.

#### 2.3.2 Think/No-Think phase

**Fig. 1:**
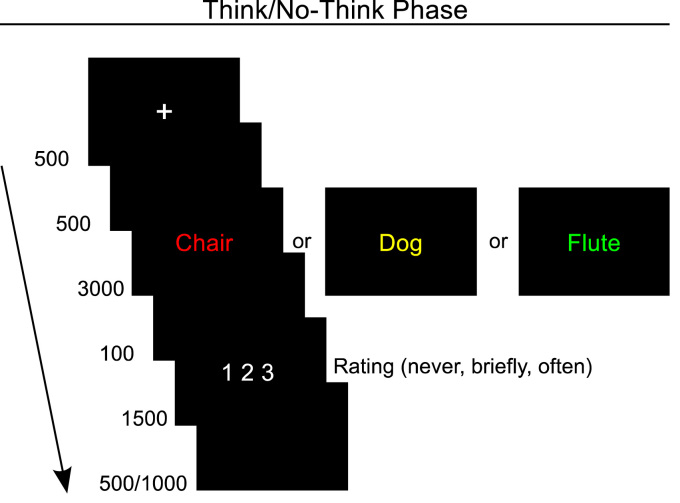
Trial structure in the Think/No-Think phase. The colour of the cue indicated if the participants should think of the associate (green; Think condition), prevent the associate from coming to mind (red; No-Think condition) or pay full attention to the cue (yellow; Perceptual baseline condition). The participants were instructed to rate whether and how often they thought of the associate on a scale from one to three (never, briefly, often) when “1 2 3″ was presented. The Presentation times are displayed in milliseconds. (For interpretation of the references to colour in this figure legend, the reader is referred to the web version of this article.)

The trial structure of the Think/No-Think phase is depicted in Fig. 1. This phase was divided into eight blocks. In the each block, 72 cues (24 per condition) were shown in green, yellow or red font at the centre of the screen. When a cue was presented in green, the task was to think of the learned associate as quickly as possible and to keep it in mind until the cue disappeared from the screen. The single words were presented in yellow font (perceptual baseline condition) and we instructed the participants to read the yellow cues and pay full attention to them until they disappeared from the screen. When a cue appeared in red (No-Think condition), the task was to prevent the associate from coming to mind. We told the participants that they still should read the cue word and pay full attention to it while trying to keep the associate from coming to mind. After each trial the participants rated the extent to which they thought of the associate on a scale from one to three (never, briefly, often) via button presses on a response box. We instructed the participants to make their ratings fast and intuitively to minimize the risk that they thought of the associate while rating. We also told the participants explicitly that they should avoid thinking of the associate while rating. The yellow single words were not associated with an associate, so we instructed the participants to rate the extent to which they thought about something else than the cue on these trials. Following the procedure described in Levy and Anderson (2012), we included three practice blocks on filler items and three structured interviews with corrective feedback (one after the first practice block, one after the second practice block and one after half of the Think/No-Think blocks) to ensure that the participants understood and followed the instructions.

#### 2.3.3 Test phase

In the final part of the experiment, we gave the participants a surprise cued recall test. The participants performed a short practice test with filler items, before the final cued-recall test began. In the final test, the cues were presented in white font colour, in random order, in the centre of the screen for 2000 ms. We instructed the participants to retrieve the learned associate and to respond orally when a question mark appeared on the screen (the question mark was presented for 2000 ms). The single words from the perceptual baseline condition were not included in this test. Importantly, we instructed the participants to recall all associates in the final test, including the ones that were related to red cues in the Think/No-Think phase.

### 2.4 EEG recording and preprocessing

The electroencephalogram was recorded continuously from 30 silver/silver chloride electrodes with a sampling rate of 1000 Hz and amplified from DC to 100 Hz with a Neuroscan (Compumedics, El Paso, TX, USA) NuAmps amplifier. The preprocessing and analysis of the EEG data was performed in Matlab (version R2014a; MathWorks, Inc, MA, USA) using the EEGlab toolbox (Delorme and Makeig, 2004), the ERPlab toolbox (Lopez-Calderon and Luck, 2014) and self-written code. The electrodes were referenced to the left mastoid during acquisition and re-referenced off-line to the average of the left and the right mastoid. Electrodes were placed above and below the left eye and at the outside of the left and the right canthi to measure the electroocculogram. Impedances were kept below 5 kΩ throughout the recording. A band-pass filter was applied offline (0.3–30 Hz, 12 decibel/octave) to increase the signal-to-noise ratio. The continuous EEG was segmented into epochs beginning 200 ms before and ending 3000 ms after the onset of the cue. The prestimulus interval was used for baseline correction of the ERPs. Ocular artefacts were corrected using the independent component analysis (ICA) procedure in the EEGlab toolbox (Delorme and Makeig, 2004). Epochs containing artefacts were rejected prior to averaging. The average number of accepted trials was 137 in the Think condition (range = 97–173, *SD* = 21), 135 in the No-Think condtion (range = 71–172, *SD* = 24) and 164 in the Perceptual baseline condition (range = 131–188, *SD* = 14). The No-Think condition was divided into memory intrusion trials and avoided retrieval trials based on the subjective ratings (Fig. 1). “Briefly” and “often” responses were coded as memory intrusions and “never” responses were coded as avoided retrievals. The mean number of accepted trials was 69 for memory intrusions (range = 20–117, *SD* = 24) and 65 for avoided retrievals (range = 14–131, *SD* = 30).

### 2.5 ERP analysis

To quantify the ERP data, we calculated mean amplitudes for each condition in seven time windows (150–250 ms, 300–450 ms, 450–550 ms, 550–900 ms, 900–1300 ms, 1300–2000 ms, 2000–3000 ms). We selected these time windows based on previous findings described above and on visual inspection of the ERP waveforms. We included the 300–450 ms time window to capture the FN400 effect, the 550–900 ms to capture the onset of the NSW and the LPP effect and the three final time windows (900–1300 ms, 1300–2000 ms and 2000–3000 ms) to capture the continuation of these effects. In addition to these memory effects, we added two additional time windows to quantify a P2 effect (150–250 ms) and an N3 effect (450–550 ms).

We divided the electrodes into nine regions of interest (left anterior: FP1, F7, F3, FC5; mid anterior: FZ, FC1, FC2; right anterior: FP2, F4, F8, FC6; left central: T7, CP5, C3; mid central: CP1, CZ, CP2; right central: C4, CP6, T8; left posterior; P7, P3, PO9; mid posterior: O1, PZ, O2; right posterior: P4, P8, PO10) and calculated mean amplitudes for each region of interest in each time window. We included the topographical factors Anterior/Posterior (anterior/central/posterior) and Hemisphere (left/midline/right) as well as a Condition factor (voluntary retrieval/perceptual baseline/memory intrusion/avoided retrieval) in an omnibus repeated measures analysis of variance (ANOVA) that we conducted in each time window, using SPSS for Macintosh version 22 (IBM Corp, Armonk, NY, USA). We used Greenhouse-Geisser correction when Mauchly's test of sphericity indicated that the data violated the sphericity assumption. Only effects involving the factor Condition were followed-up. We conducted four planned comparisons in each time window. First, we compared the voluntary retrieval and intrusions with the perceptual baseline to investigate the ERP correlates of these two processes separately. Next, we compared voluntary retrieval and memory intrusions to examine differences in temporal dynamics between these two types of retrieval. Finally, we contrasted memory intrusions with avoided retrievals within the No-Think task to investigate if the ERP effects revealed the awareness of a memory intrusion.

Next, we conducted a second set of ANOVAs with vector-scaled amplitude differences to test if the memory intrusion and the voluntary retrieval effects had distinct topographies. In these analyses, we only included memory effect time-windows encompassing significant voluntary retrieval and memory intrusion effects (as compared with the perceptual baseline; i.e. 300–450, 550–900, 900–1300 ms). We first computed vector-scaled differences for the memory intrusion and the voluntary retrieval effects to control for differences in source strength (McCarthy and Wood, 1985). Next, we performed a Voluntariness (memory intrusion–perceptual baseline baseline/voluntary retrieval–perceptual baseline)xRegion (left anterior/mid anterior/right anterior/left central/mid central/right central/left posterior/mid posterior/right posterior) repeated measures ANOVA in each time window. We restricted follow-up analyses to Voluntariness x Region interactions. In the results section, we refer to these tests of differences in topography as topographic analyses.

The FN400 and the N2 have been related to forgetting in previous studies. To examine the relationship between these ERP effects and forgetting, we calculated difference scores between the memory intrusion ERPs and the perceptual baseline ERPs in the electrode regions and the time windows where these ERP effects were significant (cf. Hellerstedt and Johansson, 2014, Waldhauser et al., 2012) and correlated theses difference scores with forgetting. Spearman's correlations were used in all correlational analyses. Given that behavioural measures of memory intrusions have been related to forgetting in previous studies (Levy and Anderson, 2012), we also correlated memory intrusion sensitive ERP effects with forgetting. To facilitate the interpretation of the direction of the correlations, the difference scores were calculated by subtracting the condition with more negative amplitude from the condition with more positive amplitude, so that a more positive value always indicated a larger ERP effect. When there was a correlation between forgetting and a memory intrusion ERP effect, we also investigated the relationship between forgetting and avoided retrieval ERP effects in the same time window to test if the relationship with forgetting was unique for memory intrusions or general for the No-Think task. Finally, when suppression-induced forgetting only correlated with ERPs from one kind of No-Think trials (i.e. only memory intrusions or only avoided retrieval) in a time window, we conducted a Meng's Z test of correlated correlation coefficients to test if there was a significant difference in correlation strength (Meng et al., 1992).

## 3 Results

We first verified that our modified Think/No-Think protocol replicated existing behavioural findings observed in the retrieval suppression literature. We then examined whether ERPs could allow us to track the time course of memory intrusions, and identify their similarities and differences from successful voluntary retrievals.

### 3.1 Behavioural results

Prior to the Think/No-Think task, the participants learned the word pairs to a high standard to ensure that sufficient intrusions would occur during the subsequent Think/No-Think task. The average learning rate was 80.3% (*SD* = 9.3%), as indicated by the criterion test. Only learned items were included in the analyses below.

#### 3.1.1 Intrusions during the Think/No-Think task

First, we examined whether intrusions occurred during the Think/No-Think task, and how they were affected by efforts to suppress retrieval. The introspective reports for each of the three conditions in the Think/No-Think phase are reported in Table 1. As evident in this table, ‘often’ responses were infrequent in the No-Think condition so the two intrusion alternatives (‘briefly’ and ‘often’) were collapsed into a single memory intrusion category in the analyses below (cf. Levy and Anderson, 2012). Based on this measure, we observed that intrusions were frequent overall, but were substantially reduced in the second as compared with the first half of the Think/No-Think phase (*F*(1,31)=57.497, *p*<.001, *η*^2^_p_=.650). Thus, with repeated effort at suppressing retrieval, the participants were able to gradually reduce the occurrence of memory intrusions (Benoit et al., 2014, Levy and Anderson, 2012).

#### 3.1.2 Memory performance in the final test

Next, we examined whether memory performance on the final recall test was affected by suppressing episodic retrieval during the Think/No-Think phase. Consistent with this possibility, average recall in the final memory test was 95% (*SD* = 9.4) in the behavioural baseline condition, 89.7% (*SD* = 8.3) in the No-Think condition, and 94.5% (*SD* = 6.9) in the Think condition. A one-way repeated measures ANOVA with the three conditions (behavioural baseline/No-Think/Think) as levels indicated a reliable main effect of Condition (*F*(2,62) = 4.951, *p* = .01; *η*^2^_p_ = .138). Replicating previous studies, planned pairwise comparisons showed that performance was lower in the No-Think condition compared with the behavioural baseline condition (*F*(1,31) *=* 6.838*, p* = .014, *η*^2^_p_ = .181), and with the Think condition (*F*(1,31) = 7.562, *p* = .010, *η*^2^_p_ = .196), i.e. there was a reliable suppression-induced forgetting effect. There was no increase in performance in the Think condition compared with the behavioural baseline condition (*F*(1,31) = .085, *p =* .772, *η*^2^_p_ = .003).

#### 3.1.3 Relationship between the reduction in memory intrusions and later suppression-induced forgetting

We examined whether the participants' ability to abate intrusions over blocks predicted the inhibitory aftereffects of suppression measured on the final recall test. First, we computed a suppression-induced forgetting index by subtracting No-Think performance from behavioural baseline performance and dividing the difference by behavioural baseline performance (to control for individual differences in behavioural baseline performance). Next, we calculated a memory intrusion slope score based on intrusion frequencies for No-Think items across the eight blocks of the Think/No-Think phase. This slope score quantified the effectiveness with which the participants down-regulated memory intrusions over repetitions. To account for variability in initial memory intrusion levels, we proportionalized the intrusion-frequency measures on intrusion frequency in the first run of the Think/No-Think phase. We then multiplied the slope measure by −1 to render positive instead of negative slope values. To control for possible memorability and intrusiveness differences between stimulus sets, both the beta slope values and the suppression score were z-normalized within each counterbalancing group (cf. Levy and Anderson, 2012).

As expected, we observed a positive correlation between memory intrusion slopes and suppression scores (*r*_s_ = .399, *p* = .024). This finding indicates that a steeper reduction in memory intrusions over repeated retrieval suppression attempts predicted greater suppression-induced forgetting, as observed in prior work (Levy and Anderson, 2012).

### 3.2 ERP results

**Fig. 2:**
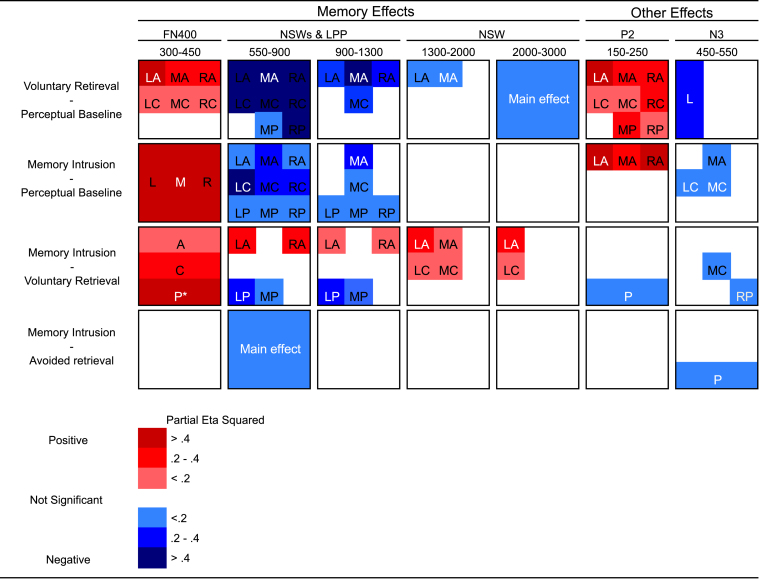
Results from follow-up analyses of significant interactions in the planned comparisons. Only significant interactions involving the factor Condition were followed up. The magnitude and the direction of the effects in each region are illustrated using a colour scale. The abbreviation is written in white type font in the regions where the effects had maximal effect size (*η*^2^_p_ ). Abbreviations: L = Left, M = Midline, R = Right, A = Anterior, C = Central, P = Posterior, LA = Left Anterior, MA = Mid Anterior, RA = Right Anterior, LC = Left Central, MC = Mid Central, RC = Right Central, LP = Left Posterior, MP = Mid Posterior, RP = Right Posterior. *There was both a Condition x Anterior/posterior and a Condition x Hemisphere interaction in the memory intrusion versus voluntary retrieval comparison in this time window. *There was both a Condition x Anterior/posterior and a Condition x Hemisphere interaction in the memory intrusion versus voluntary retrieval comparison in this time window.

In examining the electrophysiological correlates of memory intrusions, we first present the results of analyses focused on whether memory intrusion effects generate retrieval-related ERP effects that resemble voluntary retrieval. We then discuss other hypothesized ERP components of interest. Fig. 2 illustrates the results from the follow-ups of the planned comparison ANOVAs (results from the omnibus ANOVA and the four planned comparison ANOVAs are displayed in Appendix A).

#### 3.2.1 Retrieval-related ERP effects

##### 3.2.1.1 FN400

**Fig. 3:**
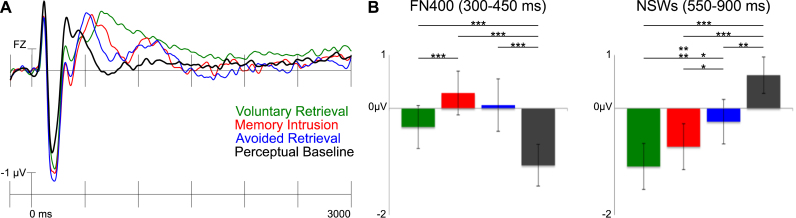
A) Grand average ERPs from the Think/No-Think phase from a Mid Anterior electrode (FZ). Negative is plotted upwards. The ERPs were filtered with a 15 Hz low-pass filter for illustrational purposes. A grid with electrodes from all nine regions of interest is presented in Appendix B. B) Bar graphs illustrating the two memory effects. Mean amplitudes (+/− standard error) from the Mid Anterior region is presented for voluntary retrieval (green), memory intrusion (red), avoided retrieval (blue) and perceptual baseline (dark grey) trials (*** = *p* < .001; ** = *p* ≤ .01; * = *p* ≤ .05). (For interpretation of the references to colour in this figure legend, the reader is referred to the web version of this article.)

**Fig. 4:**
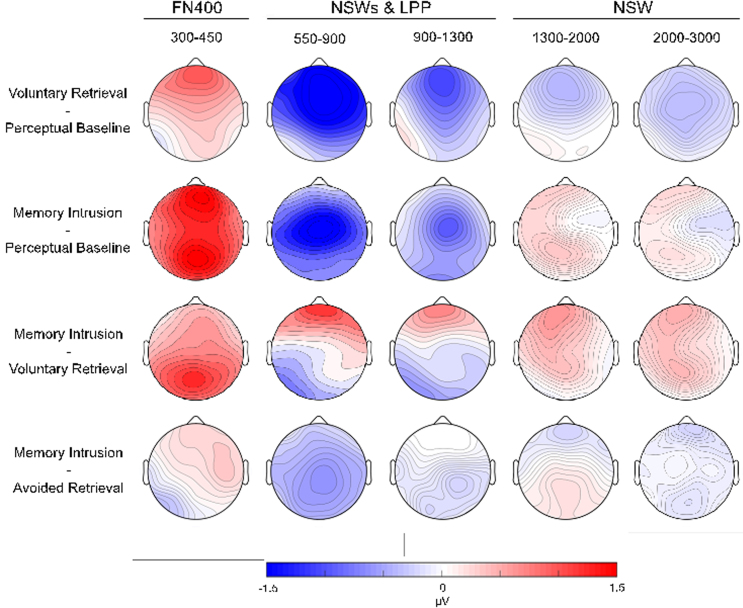
Topographical maps illustrating the scalp distribution of amplitude differences from each of the four planned comparisons in the memory effect time windows.

We expected the presentation of the cue in the Think/No-Think phase to reactivate the paired associate during both voluntary retrievals and memory intrusions, but not in the perceptual baseline condition (because the cues were not paired with associates in this condition). We expected that this reactivation would be reflected in an FN400 effect. More specifically, we predicted that both voluntary retrieval and memory intrusion ERPs would be more positive going compared with the perceptual baseline in the mid anterior region in the 300–450 ms time window. In line with these predictions, planned comparisons showed that both voluntary retrieval ERPs and memory intrusion ERPs were more positive going compared with perceptual baseline ERPs and that the effect was most pronounced over midline regions (Fig. 3, Fig. 4). Interestingly, there were no amplitude differences between memory intrusion and avoided retrieval ERPs in this time window. Thus, in line with the reactivation interpretation of the FN400, an FN400 occurred in all conditions in which the cue had been related to an associate.

Next, we examined the relationship between the FN400 memory intrusion effect and suppression-induced forgetting in the 300–450 ms time window. There was a positive correlation between forgetting and the magnitude of the memory intrusion FN400 effect (the larger the memory intrusion FN400, the more forgetting) in the mid anterior (*r*_*s*_ = .422, *p* = .016) and the right anterior region (*r*_*s*_ = .471, *p* = .006) and a borderline significant correlation in the left anterior (*r*_*s*_ = .331, *p* = .065) and the right central (*r*_*s*_ = .348, *p* = .051) regions. To investigate if forgetting was specifically related to the memory intrusion FN400 or more generally related to the FN400 in the No-Think task, we tested if there was a similar correlation between forgetting and the avoided retrieval FN400. Importantly, there was no such correlation (all *p*s≥.135), suggesting that the intrusion FN400 effect is associated with additional processes that cause forgetting. Next, we tested if the intrusion FN400 correlated more strongly with suppression-induced forgetting than the avoided retrieval FN400. This test indicated no reliable difference in correlation strength (Meng's *Z* = 1.482, *p* = .138).

Finally, as illustrated in Fig. 2, Fig. 4, there was a posterior positive going P3-like effect in the No-Think condition (observed for both memory intrusion and avoided retrieval trials) compared with the perceptual baseline condition in the same time window (300–450 ms). The topographic analysis resulted in a significant Voluntariness x Region interaction (*F*(8.248) *=* 6.567*, p* = .001, *η*^2^_p_ = .175) indicating that the voluntary retrieval and the memory intrusion effects had separate topographies in this time window. Follow-up analyses showed that the interaction was due to a greater memory intrusion effect over posterior regions (all *p*s≤.017); there was a posterior P3 effect for memory intrusions, but not for voluntary retrieval in this time window. The posterior P3 is dissociable from the anterior FN400 as only the latter correlated with suppression-induced forgetting in the memory intrusion condition and as the P3 was absent for voluntary retrieval. The posterior P3 effect may reflect allocation of visual attention to the No-Think cues that were related to the more demanding task (Polich, 2007). In line with this interpretation, there was no difference in P3 amplitude between memory intrusions and avoided retrievals indicating that the effect was general for the No-Think condition.

##### 3.2.1.2 Negative slow wave

We expected both voluntary retrieval and memory intrusions to be associated with an NSW effect compared with the perceptual baseline. Voluntary retrieval (in the Think condition) was indeed related to a sustained NSW (maximal over anterior and central regions) from 550 ms until the end of the recording epoch (see Fig. 2, Fig. 3, Fig. 4). In contrast, memory intrusions triggered an NSW effect that was restricted to the 550–900 (central maximum) and the 900–1300 ms time windows (mid anterior maximum). These findings suggest that participants purged the intruding associate out of awareness when they detected an intrusion, consistent with our instructions.

The preceding findings suggest that the NSW may index the extent to which an unwanted memory has intruded into and remained in working memory during the suppression epoch, providing a useful indirect metric of conscious memory intrusions. If correct, this interpretation predicts three key findings. First, we should find that the NSW effect should be significantly greater for intrusions than for successfully avoided retrievals and should be largely absent for the latter, given the reported absence of the item in awareness. Second, the duration of the NSW should be significantly shorter for intrusions than for voluntary retrievals, confirming the impression created by the foregoing findings. Finally, if the NSW effect indexes the time an unwanted memory remains in working memory, it should faithfully track reductions in the frequency of intrusions across the blocks of the Think/No-Think task, as intrusions will arise on fewer trials in later blocks.

We observed evidence consistent with all three of these predictions. First, we found a significantly greater NSW effect for intrusions compared with avoided retrievals in the 550–900 ms time window (Fig. 2, Fig. 3, Fig. 4). This greater NSW for intrusions is consistent with the participants’ reports that the associate entered awareness on intrusion trials, but not on successful avoided retrieval trials.

Second, the duration of the NSW for voluntary retrieval was indeed longer than it was for intrusions. To show this formally, we quantified the duration of the effect in the Think and the intrusion conditions. We first smoothed the ERP data with a 15 Hz low-pass filter. Next, we calculated difference waves by subtracting the perceptual baseline ERPs from the retrieval conditions ERPs (separately for memory intrusions and voluntary retrievals). We then detected the start and the end points of the NSW effect separately for memory intrusions and voluntary retrievals for each participant at electrode FZ (the electrode with greatest effect size for both types of retrieval). We defined the onset (and the offset) as the time point characterized by the presence (or the absence) of a negative amplitude difference that lasted for at least 50 ms (i.e. 25 samples). We used this criterion to reduce the influence of high frequency fluctuations. We considered the first sample with negative amplitude from 550 ms into the epoch (the starting point of the NSW in the grand average) that met the criterion as the start point and the first sample with non-negative (positive or zero) amplitude that met the criterion as the end point. Finally, we calculated the duration by subtracting the start point from the end point. As predicted, a repeated measures ANOVA comparing voluntary and memory intrusion NSW duration indicated that the NSW effect was shorter for memory intrusions than it was for voluntary retrieval (voluntary retrieval NSW, *M* = 909; memory intrusion NSW, *M* = 579; *F*(1,31) = 11.133, *p* = .002, *η*^2^_p_ = .264) .

Finally, we found that the NSW indeed was larger during the first half of the Think/No-Think phase compared to the second half, when contrasting the No-Think condition (collapsed over memory intrusion and avoided retrieval trials) to the perceptual baseline condition. In testing this prediction, we calculated a mean amplitude difference score between the No-Think and the perceptual baseline conditions for the first and the second half of the Think/No-Think phase in the 550–900 ms time window. Next, we conducted a Repetition (first half/second half) x Anterior/posterior (anterior/central/posterior) x Hemisphere (left/midline/right) repeated measures ANOVA. There was a main effect of Repetition (*F*(1,31) = 8.846, *p* = .006, *η*^2^_p_ = .222), a Repetition x Anterior/posterior interaction (*F*(2,62) = 4.440, *p* = .024, *η*^2^_p_ = .125) and a Repetition x Hemisphere interaction (*F*(2,62) = 7.223, *p* = .002, *η*^2^_p_ = .189). Consistent with the prediction, the NSW effect was larger during the first compared to the second half. Follow-up analyses of the Repetition x Anterior/posterior interaction showed that the effect was larger in the first half in all levels of the Anterior/posterior factor (all *p*s≤.038) and that the amplitude difference between halves was maximal over central regions (*F*(1,31) *=* 12.253, *p* = .001, *η*^2^_p_ = .283). In addition, follow-up analyses of the Repetition x Hemisphere interaction indicated that the effect was larger in the first half in left and midline regions (all *p*s ≤ .004) and that the amplitude difference between halves was maximal in midline regions (*F*(1,31) *=* 10.295, *p* = .003, *η*^2^_p_ = .249). These findings suggest that as unwanted memories became progressively less common over blocks, that NSW index grew smaller, consistent with the view that this effect could indirectly index the intrusion of unwanted memories into working memory.

A correlation between the reduction in the NSW between halves of the Think/No-Think phase and the reduction in the behavioural measure of intrusions between the two halves of the Think/No-Think phase would provide additional evidence for the link between the two measures. There was a marginally significant negative correlation between these two measures over the midcentral region (*r*_*s*_ = .335, *p* = .061), indicating that the reduction in reported memory intrusions was reflected in a reduction in the NSW.

Despite the overall consistency of the relation between the NSW and level of mnemonic awareness, the NSW for intrusions exhibits important differences from that observed for voluntary retrieval. When we directly compared the NSW across these two conditions, we found that memory intrusion ERPs were more positive going over lateral anterior regions and more negative going over left and mid posterior regions in the 550–900 and the 900–1300 ms time windows (see Fig. 4). The difference in topography of the memory intrusion and the voluntary retrieval ERP effects was confirmed by the topographic analysis. There was a robust Voluntariness x Region interaction in both the 550–900 and the 900–1300 ms time window (all *p*s<.001), showing that the two effects had separate topographies in these time windows. Follow-up analyses showed that the voluntary retrieval NSW effect was greater than the memory intrusion NSW over lateral anterior regions (all *p*s≤.049). In addition, the same analyses also showed that the memory intrusion ERPs were more negative going over left and mid posterior regions (all *p*s ≤ .034), indicating that there was an LPP effect in the voluntary retrieval condition that was reduced for memory intrusions (the LPP effect is described in Section 3.2.1.4.). In sum, voluntary retrieval gave rise to a more anteriorly distributed NSW as compared with memory intrusions.

##### 3.2.1.3 Relation between the NSW and suppression-induced forgetting

Although both the FN400 and the NSW broadly can be viewed as indexing memory reactivation, the NSW observed here selectively couples with reports of phenomenal awareness, whereas the FN400 does not (being present even for avoided retrieval). This raises the question of whether increases in the NSW predict increased forgetting, as does the FN400, or whether intrusion into working memory alters the relationship to forgetting. Strikingly, we found that, in contrast to the FN400, the memory intrusion NSW correlated negatively with forgetting (the larger the NSW the less forgetting) in the 550–900 ms time window in the left anterior region (*r*_*s*_ = −.391, *p* = .027). In addition, there were marginally significant correlations between the same effects in the mid anterior (*r*_*s*_ = −.345, *p* = .053) and the right anterior region (*r*_*s*_ *=* −.335, *p* = .061). There was no such correlation between amplitude differences between avoided retrieval and the perceptual baseline ERPs in this time window (all *p*s≥.138), indicating that the correlation was specific for the memory intrusion NSW. Next, we tested if the intrusion NSW correlated more strongly with suppression-induced forgetting than the avoided retrieval NSW with Meng's Z test of correlated correlation coefficients. This test revealed no reliable difference in correlation strength (*Z* = −.793, *p* = .428).

##### 3.2.1.4 Left parietal positivity

Based on prior work linking both the NSW and the LPP to retrieval processes, we had hypothesized that both these effects would be present whenever awareness of a memory was reported. By this view, the LPP, like the NSW, should be present both for voluntary retrievals and for memory intrusions. Specifically, we predicted that these conditions would be more positive going compared with the perceptual baseline condition in the left posterior region approximately 500 ms after the presentation of the cue.

**Fig. 5:**
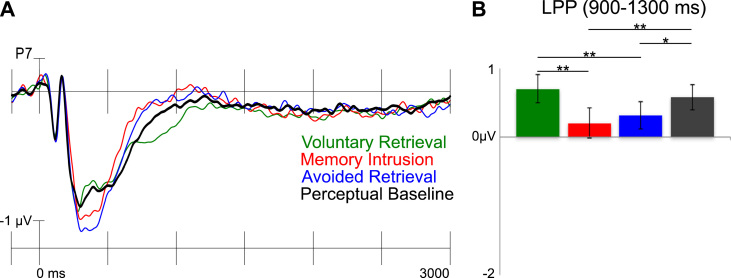
A) Grand average ERPs from the Think/No-Think phase from a Left Posterior electrode (P7). Negative is plotted upwards. The ERPs were filtered with a 15 Hz low-pass filter for illustrational purposes. B) Bar graph illustrating the LPP effects. Mean amplitudes (+/− standard error) from the Left Posterior region is presented for voluntary retrieval (green), memory intrusion (red), avoided retrieval (blue) and perceptual baseline (dark grey) trials (*** = *p* < .001; ** = *p* ≤ .01; * = *p* ≤ .05). (For interpretation of the references to colour in this figure legend, the reader is referred to the web version of this article.)

Unexpectedly, however, there was no difference in ERP amplitude between voluntary retrieval and perceptual baseline ERPs in the left posterior region in the 550–900 or the 900–1300 ms time windows, indicating that there was no LPP effect. Previous studies that have reported a reduction in the LPP after retrieval suppression have not included a perceptual baseline condition and have instead contrasted the Think and the No-Think conditions (Bergström et al., 2007, Bergström et al., 2009, Bergström and de Fockert, 2009, Chen et al., 2012, Hanslmayr et al., 2009, Mecklinger et al., 2009, Waldhauser et al., 2012). When comparing voluntary retrieval and avoided retrieval ERPs, there was an LPP effect in the left posterior region in the 900–1300 ms time window (see Fig. 5; there was also a trend for the same effect in the 550–900 ms time window, *p* = .058). We next examined whether memory intrusions also were related to a similar LPP effect, as would be expected if the LPP effect indexed mnemonic awareness. Contrary to our prediction, there was no LPP effect for memory intrusions in comparison with avoided retrieval (there was even a trend towards more negative going ERPs for memory intrusions in the left posterior region in the 550–900 ms time window, *p* = .058). In fact, similar to avoided retrieval there was a reduction in the LPP for memory intrusions compared with voluntary retrievals in both the 550–900 and the 900–1300 ms time windows (all *p*s≤.002; see Fig. 2, Fig. 5). The LPP was reduced for intrusions even when compared with the perceptual baseline (see Fig. 5).

#### 3.2.2 Other ERP effects

##### 3.2.2.1 P2

As expected, both voluntary retrieval and memory intrusions were related to a greater P2 as compared with the perceptual baseline condition in the 150–250 ms time window. These P2 effects showed maximum amplitude over anterior regions. There was no difference in P2 amplitude between memory intrusion and avoided retrieval ERPs. The voluntary retrieval ERPs were more positive going over posterior regions when compared with memory intrusion in the 150–250 ms time window, but this was due to component overlap, rather than a greater P2 (see Fig. 2 and Appendix B). The P2 effect may reflect that the participants allocated more attention to green and red cues, in the Think and the No-Think conditions, compared with the perceptual baseline condition.

##### 3.2.2.2 N3

Previous studies have reported greater N2 (e.g. Bergström et al., 2009, Mecklinger et al., 2009, Waldhauser et al., 2012) and N3 (Waldhauser et al., 2012) amplitude in the No-Think condition compared with the Think condition. In the present study, we observed an N3-effect, but no N2 effect. More specifically, N3 amplitude was greater for memory intrusions compared with the perceptual baseline condition over central regions in the 450–550 ms time window (Fig. 2). This effect was larger for memory intrusions compared with the perceptual baseline condition (significant in mid anterior, left central and mid central regions) and the voluntary retrieval (significant in the mid central and the right posterior region). Unexpectedly, memory intrusion ERPs were more negative than avoided retrieval ERPs in posterior regions. This effect may indicate the onset of the NSW that becomes more widespread in the subsequent time window. The N3 peak has previously been positively related to forgetting in one Think/No-Think study (Waldhauser et al., 2012), but there was no correlation between the N3 and forgetting in the present study (all *p*s≥.151).

Taken together we replicated the P2 and the N3 effects that prior retrieval suppression studies have observed. Given that we focus on retrieval effects in the present study and that the P2 and N3 replicate previous observations without offering new information regarding their functional significance, we will not discuss these two effects further.

## 4 Discussion

Memory intrusions constitute a core symptom of several psychiatric disorders. To our knowledge, the present study is the first to investigate the temporal dynamics of memory intrusions. The high temporal resolution of ERPs combined with introspective reports made it possible to track the involuntary intrusion of unwanted memories into awareness. We focused on three ERP memory effects, the FN400, the NSW and the LPP that have been related to processes involved in voluntary retrieval and identified differences and similarities between memory intrusions and voluntary retrievals in relation to these effects. Before discussing the ERP results, we will briefly highlight the behavioural results.

On the whole, we replicated the main findings typically observed in studies of retrieval suppression. As expected, repeated retrieval suppression attempts during the Think/No-Think phase caused suppression-induced forgetting on the final test (e.g. Anderson et al., 2004, Anderson and Green, 2001). Moreover, consistent with prior studies that have collected phenomenological reports of intrusions, intrusion frequency declined substantially with repeated suppression in the Think/No-Think phase (Benoit et al., 2014, Levy and Anderson, 2012) and this reduction predicted ensuing suppression-induced forgetting: the steeper the reduction in intrusion frequency, the worse memory for suppressed items was, relative to behavioural baseline items, on the later test (Levy and Anderson, 2012).

One finding that diverges from some earlier studies was that repeated retrieval of Think items in the Think/No-Think phase did not enhance later recall compared with performance in the behavioural baseline condition. This lack of facilitation may have arisen from changes to the learning procedure that we adopted specially for this study. To obtain enough memory intrusions to enable ERP analyses, we set our learning criterion higher in the present study compared to studies that have showed such an increment (e.g. Anderson and Green, 2001). Increasing the learning threshold caused near ceiling performance in the final recall test in both the behavioural baseline and the Think conditions, likely contributing to the lack of facilitation in the latter condition.

Taken together, the behavioural results confirmed that our modified version of the Think/No-Think paradigm replicated key findings observed in the retrieval suppression literature. Critically, the intrusion reporting procedure adopted here allowed us to isolate trials in which involuntary retrieval occurred, laying the groundwork for our efforts to use ERP analysis to track the online dynamics of unwanted memories intruding into awareness.

### 4.1 The temporal dynamics of memory intrusions

To isolate the online dynamics of memory intrusions, our strategy was to compare ERPs observed during intrusions with those observed during voluntary retrievals (i.e., Think trials) and also during avoided retrievals. Here we discuss key similarities and differences between intrusions and voluntary retrievals for each of the three key memory related ERP effects: the FN400, the Negative Slow Wave, and the Left Parietal Positivity. We then offer a discussion characterizing the implications of these findings for understanding the processes involved in progressing from cue presentation to memory retrieval.

#### 4.1.1 The FN400 reflects memory reactivation and the need for cognitive control

The FN400 was attenuated for both voluntary retrieval and intrusion trials, compared with that observed during the perceptual baseline condition (Fig. 3, Fig. 4). The cues in each of these conditions were paired with associates, but they were not in the perceptual baseline condition, so the reduced FN400 (i.e., the FN400 effect) may reflect reactivation of the associates, consistent with prior evidence for the role of this component in memory reactivation (Hellerstedt and Johansson, 2014, Opitz and Cornell, 2006).

Hellerstedt and Johansson (2014) showed that during competitive retrieval, the magnitude of the FN400 observed for reactivated competing memories predicted greater retrieval-induced forgetting for those competitors on a later test. We found an analogous effect in the present study, wherein the memory intrusion FN400 effect was positively correlated with later suppression-induced forgetting (i.e., the larger the FN400 effect for intrusions, the more suppression-induced forgetting). These findings suggest that the FN400 effect may index memory reactivation that is instrumental to triggering inhibitory control processes to prevent the associate from entering awareness. Interestingly, we observed no corresponding correlation between suppression-induced forgetting and the FN400 elicited during avoided retrievals, suggesting that for reactivation to lead to forgetting, an intrusion must occur. Although the difference in correlation magnitude across intrusion and avoided retrieval trials was not reliable for this FN400 measure, there is precedent for supposing that intrusions and avoided retrievals truly differ in the processes that they elicit during memory control. For instance, using the current trial-by-trial intrusion report procedure, fMRI-studies of retrieval suppression have found greater engagement of right dorsolateral prefrontal cortex during intrusions and stronger down-regulation of retrieval related activity in the hippocampus (Benoit et al., 2014, Levy and Anderson, 2012). Indeed, whereas intrusion-related down-regulations in the hippocampus robustly predict later suppression-induced forgetting, hippocampal activations during avoided retrievals do not (Levy and Anderson, 2012). The current results converge with those findings in highlighting the critical role of intrusions in triggering inhibitory control, and further reveal that memory reactivation can be measured in EEG approximately 300 ms after the appearance of the retrieval cue (see Johansson et al., 2007 for a comparable temporal characteristic).

In ERP studies of recognition memory, the FN400 has been suggested to reflect familiarity (e.g. Rugg and Curran, 2007) or conceptual priming (Paller et al., 2007). It is unknown whether the functional accounts of the FN400 from recognition studies generalize to cued recall. First, in recognition memory the retrieval cue is a copy cue, whereas the present results showed that the cued-recall FN400 reflects the retrieval of the associated items rather than processing of the cue per se, since the FN400 was only present in conditions that included an associate (i.e. the Think and the No-Think conditions). Second, a recognition memory task assesses explicit memory retrieval, but the present cued-recall FN400 was insensitive to mnemonic awareness and observed also for avoided retrieval trials, indicating that it reflects preconscious, implicit memory reactivation. Taken together, we believe that the present FN400 effect reflects the preconscious reactivation of episodic associations (perceptual, conceptual or lexical) formed during the encoding phase and elicited by the retrieval cue. If this interpretation is correct, the FN400 effect for avoided retrievals shows that the associate can be activated without a concomitant intrusion, and that an additional (and likely later) process is needed to explain how intrusions and avoided retrievals differ. The first evidence for a differentiation of trials according to reported mnemonic awareness was detected in the negative slow wave, which we describe next.

#### 4.1.2 Negative slow waves index the presence of retrieved memories in working memory

Over the recording epoch for each trial, the first reliable indication of a distinction amongst trials according to mnemonic awareness was the NSW. Consistent with prior work associating this component with working memory maintenance (Drew et al., 2006, Ruchkin et al., 2003), voluntary retrieval and maintenance of an item throughout Think trials was reflected in an enhanced NSW. Interestingly, however, intrusion trials also triggered an NSW, suggesting that this component is sensitive to mnemonic awareness, but does not require intentional retrieval or active maintenance to arise. Consequently, the NSW could, intriguingly, index the intrusion of an unwanted memory into working memory.

Confirming this possibility, the NSW was (a) greater for intrusions compared with avoided retrievals in the 550–900 ms time window, (b) shorter for intrusions compared with voluntary retrievals of Think items, as one might expect if participants sought to limit awareness of intrusive memories, and (c) smaller for No-Think trials in the second half of the Think/No-Think phase, during which intrusion trials were significantly less frequent. These findings suggest that we could detect the brief emergence of an intrusive memory into working memory, and its rapid purging, consistent with our instructions to exclude the No-Think items from awareness. If so, they provide converging evidence that people grew more successful at controlling mnemonic awareness throughout the Think/No-Think phase.

Rehearsal in working memory is typically thought to promote the transfer of material into long-term memory improving later memory performance. If the NSW reflects activation of an item in working memory, the magnitude of the NSW might be expected co-vary with later remembering. Strikingly, this is exactly what we found, corroborating the working memory interpretation of the NSW effect. The finding that the intrusion NSW predicted less suppression-induced forgetting suggests that whereas intrusions may trigger cognitive control processes necessary to disrupt retention, the longer an intrusion persists in awareness (as indexed by the NSW), the less effective those processes are in disrupting retention.

The NSW effect was, however, more anterior for voluntary retrieval than for memory intrusions (see Fig. 2, Fig. 3). This difference may be related to findings observed in fMRI studies that have reported increased dorsolateral prefrontal cortex activity for intentional compared with incidental retrieval (Hall et al., 2014, Kompus et al., 2011). Although we do not know the source of the NSW effect, the more anterior distribution of the voluntary retrieval NSW could plausibly arise from additional prefrontal engagement in active, intentional maintenance of associates. This topographical difference also suggests a complementary interpretation, namely that the NSW also may reflect error-detection. The error-related negativity ERP component typically shows a maximum over mid central regions (Gehring et al., 1993), similar to the memory intrusion NSW in the present study. A similar, but more posterior ERP effect, the late posterior negativity (LPN) has been associated with action monitoring in retrieval tasks that are characterized by a high error-rate (Johansson and Mecklinger, 2003), an aspect that certainly characterises the No-Think task used here. Of relevance for the present study, the LPN recently has been demonstrated in a demanding semantic cued recall task, presumably reflecting continuous error-detection (Hellerstedt and Johansson, 2016). It is conceivable that the memory intrusion NSW reflects participants detecting the error of counter-intentional retrieval. The working memory and the error-detection interpretations of the memory intrusion NSW are complementary rather than exclusive, and both processes are likely to operate during counter-intentional memory retrieval.

#### 4.1.3 The LPP is reduced during retrieval suppression attempts

Replicating previous studies, the LPP was attenuated during retrieval suppression in comparison to voluntary retrieval. In contrast with expectations, this reduction in the LPP was not only present for avoided retrievals, but also for memory intrusions, indicating that reduced LPPs do not necessarily reflect the failure of an unwanted memory to enter awareness during retrieval suppression. The LPP has been related to recollection in previous studies, but there was unexpectedly no LPP effect in the contrast between voluntary retrieval and the perceptual baseline in the present study, which would have been expected if the LPP reflects recollection of the associate. However, the LPP may not only reflect recollection of the associate, but also recollection of the cue bound to other episodic details in the encoding context. Since all cues were presented in the same way in the encoding phase, retrieval of such episodic details should be comparable for cues in all conditions, including the perceptual baseline condition. This could potentially explain the absence of LPP amplitude differences between voluntary retrieval and the perceptual baseline. The interpretation that the LPP may covary with recollection of the cue and not only with retrieval of the associate is consistent with recent cued recall studies reporting that successful associative retrieval is evident in anterior positive slow waves rather than in LPPs (e.g. Hellerstedt and Johansson, 2016). The reduced LPP for memory intrusions and for avoided retrievals suggests that retrieval suppression may reduce recollective processing of both cue and associate, and that suppression may occur even when the associate intrudes into awareness. We note that any conclusive account of the LPP during retrieval suppression awaits future experimental work designed for this purpose.

#### 4.1.4 From cue presentation to memory intrusion: integrating the findings

Taken together, the present results suggest that multiple processes are elicited in the progression from a retrieval cue to memories entering awareness. Table 2 summarizes key findings in relation to two ERP effects that index retrieval-related processes.

The earliest suggestion of a retrieval related process in our data is the putative reactivation of the associate, as signified by the FN400 effect. Given that the FN400 effect did not vary as a function of whether a memory intruded into awareness or not, and given it’s early timing, we suggest that this effect reflects a preconscious retrieval of conceptual or lexical representations, operating in a manner akin to priming (Hellerstedt and Johansson, 2014). The finding that the early reactivation was not related to later intrusion status is in line with Cowan's embedded-processes model of working memory, which holds that reactivation of a long-term memory trace is necessary, but not sufficient for causing entrance of a long-term memory trace into awareness (Cowan, 1999). Because increases in the magnitude of this FN400 effect on intrusion trials predicted suppression-induced forgetting, we suggest that this preconscious activation may index the need for inhibitory control. If the unfolding retrieval is not successfully halted or suppressed, this early reactivation will be followed by the entrance of the trace in working memory, presumably indicated by the NSW effect.

Like the FN400, the NSW also appears to index memory reactivation, but at a later point in time. Interestingly, however, although both the early reactivation and the working memory activation broadly reflect memory retrieval, they appear to be functionally distinct. We observed a double dissociation between the two ERP effects that index these processes (Table 2). First, whereas the NSW was related to mnemonic awareness of memory intrusions, the FN400 was not. Second, whereas FN400 magnitude was related to increased forgetting, NSW magnitude was related to reduced forgetting. That increasing reactivation predicts greater forgetting whereas increasing working memory activation predicts better remembering fits well with the hypothesized *demand-success trade-off* relationship between interference and inhibition described in inhibition theories (e.g., Anderson and Levy, 2010). These theories hold that increasing interference caused by unwanted traces should raise the likelihood of engaging inhibitory control processes critical to inducing forgetting of those traces; but critically, because inhibition is not always successful, the very highest levels of reactivation/interference may be associated with failed inhibition, and, consequently, later remembering of the unwanted trace. In the present study, greater early reactivation as indexed by the FN400 may signal an increased level of reactivation and a greater need for inhibition, whereas working memory activation may indirectly index the failing of inhibition. Presumably, the longer a NSW lasts on intrusion trials, the longer the unwanted item endures in working memory, reflecting a failure to suppress the reactivated memory, and an increased chance of re-encoding of the unwanted trace.

The finding that the participants indicated that the memory entered awareness, but showed no LPP (both for voluntary retrievals and memory intrusions), the putative ERP marker of recollection, suggests that associates can enter awareness in the absence of recollection. Although this result may seem surprising, successful associative retrieval without an LPP has been reported in recognition memory studies of unitization. A growing body of research suggests that when paired associates are unitized into a single representation, associative memory for the pairs depends more on familiarity-based processes mediated by the perirhinal cortex than on recollective processes supported by the hippocampus (for reviews Mayes et al., 2007, Murray, 2013). Thus, the present FN400 effect may reflect the reactivation of the unitized memory representations, explaining how the associates could enter working memory and intrude into awareness in the absence of recollection.

The present study represents a promising first step towards developing methods for tracking the intrusion of unwanted memories into awareness. Critically, neural markers of memory intrusions, like the intrusion-NSW effect, could, in principle, be harnessed to study involuntary retrieval processes in psychiatric populations suffering from intrusive memories. Recent evidence has confirmed that behavioural and electrophysiological indices of retrieval suppression in procedures highly similar to the one used here predict participants’ everyday perceptions of their memory control ability (Küpper et al., 2014), the severity of intrusion symptoms in post-traumatic stress disorder (Catarino et al., 2015), ruminative tendencies (Fawcett et al., 2015), and the frequency of distressing intrusions elicited by a traumatic film (Streb, Mecklinger, Anderson, Lass-Hennemann, & Michael, 2016). Given these findings, the intrusion markers reported here may aid in evaluating clinical interventions aiming at reducing memory intrusions in psychiatric populations. Beyond identifying a neural marker of intrusive memories, this study informs basic cognitive theories of memory by elucidating how multiple retrieval processes set-off by a reminder cue can be modulated by the intention to retrieve or to avoid retrieval.

## Figures and Tables

**Table A.1:** Results from the omnibus and the planned comparison ANOVAs in the selected time windows. The *α*-level was set to .05.

	Memory Effects					Other Effects	
	FN400	NSWs				P2	N3
Comparisons	300-450	550-900	900-1300	1300–2000	2000–3000	150-250	450-550
**Omnibus ANOVA**							
Condition	<.001	<.001	.034	NS	NS	<.001	NS
Condition x Anterior/posterior	.050	<.001	.001	.023	NS	<.001	.008
Condition x Hemisphere	<.001	<.001	.003	NS	NS	NS	.037
Condition x Aterior/posterior x Hemisphere	NS	<.001	<.001	.002	.001	.006	<.001

**Voluntary Retrieval vs Perceptual Baseline**							
Condition	.001	<.001	.004	.284	.030	<.001	NS
Condition x Anterior/posterior	.004	<.001	.003	.013	NS	.014	NS
Condition x Hemisphere	NS	<.001	<.001	NS	NS	.043	.011
Condition x Aterior/posterior x Hemisphere	.011	<.001	<.001	.005	NS	.005	NS

**Memory Intrusion vs Perceptual Baseline**							
Condition	<.001	<.001	.019	NS	NS	.010	.050
Condition x Anterior/posterior	NS	.037	NS	NS	NS	<.001	NS
Condition x Hemisphere	<.001	.001	.002	NS	NS	NS	.040
Condition x Aterior/posterior x Hemisphere	NS	<.001	.004	.014	NS	<.001	<.001

**Memory Intrusion vs Voluntary Retrieval**							
Condition	<.001	.593	NS	NS	NS	NS	.138
Condition x Anterior/posterior	.045	<.001	<.001	.044	NS	.009	NS
Condition x Hemisphere	.024	<.001	NS	NS	.083	NS	.011
Condition x Aterior/posterior x Hemisphere	NS	<.001	<.001	.001	.005	NS	.007

**Memory Intrusion vs Avoided Retrieval**							
Condition	NS	.016	NS	NS	NS	NS	NS
Condition x Anterior/posterior	NS	NS	NS	NS	NS	NS	.008
Condition x Hemisphere	NS	NS	NS	NS	NS	NS	NS
Condition x Aterior/posterior x Hemisphere	.028	NS	NS	NS	NS	NS	NS

**Fig. B.1:**
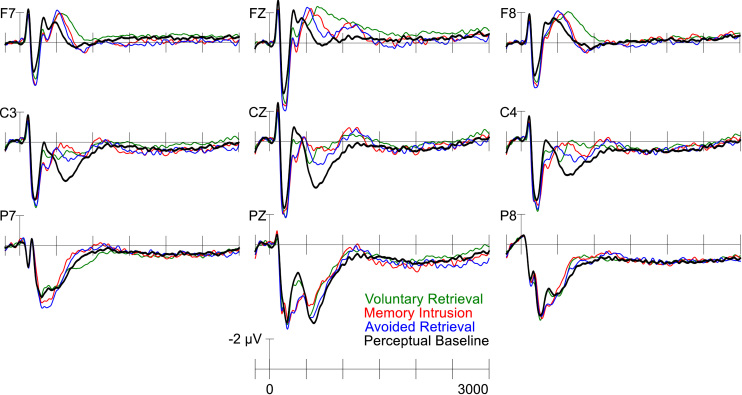
ERP amplitudes during the Think/No-Think phase. Grand average ERPs from one representative electrode from each of the nine regions of interest that were used in the statistical analyses are depicted. For illustrational purposes, the ERPs were filtered with a 15 Hz low-pass filter. Negative is plotted upwards.

**Table 1:** Introspective reports from the Think/No-Think phase. Average proportions are shown for each response separately for each of the three conditions. Standard deviations are shown in brackets.

Condition	Never	Briefly	Often
Think	6.2% (6.3)	10.6% (9.6)	83.2% (14)
No-Think	50.2 (19.5)	43.6% (16.4)	6.3% (7.4)
Perceptual baseline	93.7% (8.7)	5.2% (7.4)	.9% (2.3)

**Table 2:** Summary of key findings. The top two rows concern whether the ERP effects were observed for voluntary retrieval and memory intrusions compared with the perceptual baseline. Positive/negative indicates the direction of the correlations with suppression-induced forgetting. The third row concerns effects in contrasts between memory intrusions and avoided retrievals, indicating that the effect was related to the presence of the intruding memory in working memory.

	FN400	NSWs
**Voluntary Retrieval**	Yes	Yes, sustained
**Memory Intrusion**	Yes	Yes, truncated
Relation to Forgetting	Positive	Negative
Mnemonic Awareness	No	Yes

## Appendix A

See: Table A.1.

## Appendix B

See: Fig. B.1.
